# Genetic variation of the *RASGRF1* regulatory region affects human hippocampus-dependent memory

**DOI:** 10.3389/fnhum.2014.00260

**Published:** 2014-04-29

**Authors:** Adriana Barman, Anne Assmann, Sylvia Richter, Joram Soch, Hartmut Schütze, Torsten Wüstenberg, Anna Deibele, Marieke Klein, Anni Richter, Gusalija Behnisch, Emrah Düzel, Martin Zenker, Constanze I. Seidenbecher, Björn H. Schott

**Affiliations:** ^1^Department of Behavioral Neurology and Department of Neurochemistry and Molecular Biology, Leibniz Institute for NeurobiologyMagdeburg, Germany; ^2^Otto von Guericke UniversityMagdeburg, Germany; ^3^Department of Clinical Psychology, University of SalzburgSalzburg, Austria; ^4^Bernstein Center for Computational Neuroscience, Humboldt UniversityBerlin, Germany; ^5^Institute of Cognitive Neurology and Dementia Research, Otto von Guericke UniversityMagdeburg, Germany; ^6^Department of Psychiatry, Charité Universitätsmedizin BerlinBerlin, Germany; ^7^Department of Genetics, Radboud University Nijmegen Medical CenterNijmegen, Netherlands; ^8^Helmholtz Center for Neurodegenerative DiseasesMagdeburg, Germany; ^9^Center for Behavioral Brain SciencesMagdeburg, Germany; ^10^Department of Human Genetics, Otto von Guericke UniversityMagdeburg, Germany; ^11^Department of Neurology, Otto von Guericke UniversityMagdeburg, Germany

**Keywords:** *RASGRF1*, hippocampus, episodic memory, genetic variation, fMRI

## Abstract

The guanine nucleotide exchange factor *RASGRF1* is an important regulator of intracellular signaling and neural plasticity in the brain. *RASGRF1*-deficient mice exhibit a complex phenotype with learning deficits and ocular abnormalities. Also in humans, a genome-wide association study has identified the single nucleotide polymorphism (SNP) rs8027411 in the putative transcription regulatory region of *RASGRF1* as a risk variant of myopia. Here we aimed to assess whether, in line with the *RASGRF1* knockout mouse phenotype, rs8027411 might also be associated with human memory function. We performed computer-based neuropsychological learning experiments in two independent cohorts of young, healthy participants. Tests included the Verbal Learning and Memory Test (VLMT) and the logical memory section of the Wechsler Memory Scale (WMS). Two sub-cohorts additionally participated in functional magnetic resonance imaging (fMRI) studies of hippocampus function. 119 participants performed a novelty encoding task that had previously been shown to engage the hippocampus, and 63 subjects participated in a reward-related memory encoding study. *RASGRF1* rs8027411 genotype was indeed associated with memory performance in an allele dosage-dependent manner, with carriers of the T allele (i.e., the myopia risk allele) showing better memory performance in the early encoding phase of the VLMT and in the recall phase of the WMS logical memory section. In fMRI, T allele carriers exhibited increased hippocampal activation during presentation of novel images and during encoding of pictures associated with monetary reward. Taken together, our results provide evidence for a role of the *RASGRF1* gene locus in hippocampus-dependent memory and, along with the previous association with myopia, point toward pleitropic effects of RASGRF1 genetic variations on complex neural function in humans.

## Introduction

The Ras pathway (Ambrosini et al., 2000; Reichardt, 2006) is a vital intracellular signaling cascade implicated in learning and memory processes. Ras is involved in the extracellular signal-regulated kinase (ERK) pathway, a mitogen-activated protein kinase (MAPK) cascade (Tartaglia and Gelb, 2010) that is crucial for the expression of long-term plasticity and memory in the adult CNS (Brambilla et al., 1997; Giese et al., 2001).

Ras activation is controlled by inhibitory GTPase-activating proteins (GAPs) and activating guanine nucleotide exchange factors (GEFs) (Mizuno-Yamasaki et al., 2012). The Ras-specific guanine nucleotide-releasing factor 1 (*RASGRF1*) belongs to the GEF family and catalyzes the exchange of GDP by GTP (Zippel et al., 1997). *In vitro* and *in vivo* studies of synaptic plasticity and memory in *RASGRF1*-deficient mice have demonstrated deficits in learning and memory (Brambilla et al., 1997; Giese et al., 2001), including impaired hippocampus-dependent learning (Giese et al., 2001). Furthermore, inactivation of *RASGRF1* has been associated with a pronounced impairment of hippocampal LTD (Li et al., 2006) and LTP (Silingardi et al., 2011) and with extensive changes in neural gene expression affecting multiple developmental and plasticity pathways (Fernandez-Medarde et al., 2007).

*RASGRF1* is expressed in the murine hippocampus, neocortex, and thalamus (Brambilla et al., 1997), but also at high levels in the retina (Zippel et al., 1997). In addition to the observed memory deficits *RASGRF1*-deficient mice display a prominent ocular phenotype with photoreception deficiencies (Fernandez-Medarde et al., 2009) and heavier crystalline lenses (Hysi et al., 2010). In humans, a genome-wide association study (GWAS) identified single nucleotide polymorphisms (SNPs) in the *RASGRF1* gene region on Chr 15q25 to be associated with myopia and refractive error (Hysi et al., 2010). The strongest association was observed for the T allele of rs8027411, a variant located 78 kb upstream of the *RASGRF1* coding region, overlapping with the *RASGRF1* transcription initiation site. This association of the *RASGRF1* gene with myopia was confirmed in a genome-wide meta-analysis that included 27 studies (Verhoeven et al., 2013).

Given the combined ocular and hippocampal phenotype of *RASGRF1*-deficient mice and the association of rs8027411 with human refractive error, we hypothesized that genetic variation of *RASGRF1* may be associated with interindividual variability of learning and memory in healthy humans. Since the hippocampus plays a pivotal role in human episodic memory and since previous investigations have already identified associations of memory performance and hippocampal function with several genetic polymorphisms within or near genes encoding elements of the Ras signaling cascade, including neurotrophic (BDNF rs6265: Egan et al., 2003) and monoaminergic (5-HT2A rs6314: de Quervain et al., 2003; COMT rs4680: de Frias et al., 2004; Bertolino et al., 2006) pathways, we focused on hippocampus-dependent processes. We investigated the impact of *RASGRF1* SNP rs8027411 on human hippocampus-dependent learning and memory using well-established episodic memory tasks in two independent cohorts of young, healthy participants. Functional magnetic resonance imaging (fMRI) was performed to assess the effects of *RASGRF1* rs8027411 on hippocampal activation during episodic encoding of novel stimuli (Duzel et al., 2011) and during reward-dependent memory encoding (Wittmann et al., 2005; Krebs et al., 2009). Given previously reported associations between myopia and high intelligence (Czepita et al., 2008), we tentatively hypothesized that the T allele might exert a beneficial effect on memory performance.

## Materials and methods

We investigated the potential effect of *RASGRF1* in two behavioral and two fMRI studies using different episodic memory encoding tasks. All participants gave written informed consent in accordance with the Declaration of Helsinki and received financial compensation for participation. The work was approved by the Ethics Committee of the University of Magdeburg, Faculty of Medicine.

### Genotyping

Genomic DNA was extracted from EDTA-anticoagulated venous whole blood using the GeneMole automated DNA extraction system (Mole Genetics, Lysaker, Norway) according to the manufacturer's protocol. Genotyping of *RASGRF1* rs8027411 was performed using the restriction fragment length polymorphism method, consisting of a PCR followed by allele-specific restriction cutting. The resulting DNA fragments were separated on an ethidium bromide-stained agarose gel and visualized under UV light. Details of the genotyping protocol are displayed in Supplementary Table S1.

### Samples

#### Participants of the first cohort

The participants were recruited from a cohort investigated at the Department of Psychology, University of Magdeburg, described in detail previously (Richter et al., 2011). From this cohort, 355 young (age mean ± *SD*: 22.96 ± 2.93 years), healthy volunteers participated in at least one of the behavioral tasks. The final behavioral study sample consisted of 348 volunteers in the logical memory subscale of the Wechsler Memory Scale (WMS), and 316 volunteers in the Verbal Learning and Memory Test (VLMT). The sample size differences between the initial sample and the analyzed sample can be explained by the fact that only complete records were included in the analysis of the behavioral experiment.

#### Participants of the second cohort

The cohort consisted of 719 young (age mean ± *SD*: 23.77 ± 2.76 years), healthy volunteers of a large-scale behavioral genetic study conducted at the Leibniz Institute for Neurobiology, Magdeburg. Based on the assumption that a possibly small effect of genetic variations may not only require a large number of volunteers but also a strict control of non-genetic factors (Lee et al., 2007; Richter et al., 2013), participants were carefully screened for exclusion criteria. All participants were genetically unrelated. Educational background was homogenous within the cohort, and all participants had obtained university entrance diploma (Abitur). Importantly, all participants had undergone routine clinical interview to exclude present or past neurological or psychiatric illness, alcohol or drug abuse, use of centrally acting medication, and the presence of psychosis or bipolar disorder in a first-degree relative. Given the reported association between myopia and *RASGRF1* polymorphism, ametropia was assessed. The final behavioral study sample consisted of 575 volunteers in the logical memory subscale of the Wechsler memory scale (WMS), and 573 volunteers in the (VLMT). All participants who completed the WMS logical memory subscale and VLMT were native speakers of German. 616 volunteers answered the questions regarding ametropia. Also here, the sample size differences between the initial sample and the analyzed sample result from the fact that only complete records were included in the analysis of the behavioral experiment.

#### Participants of the fMRI experiment 1

119 young (age mean ± *SD*: 24.35 ± 2.60), healthy students from the second cohort were recruited for participation. Participants were right-handed according to self-report and reported no history of neurological or psychiatric illness, or use of any centrally acting drugs, and had T1-weighted MR images without pathological findings.

#### Participants of the fMRI experiment 2

63 young (mean age ± *SD*: 24.53 ± 2.77 years), healthy students from the second cohort were recruited for participation. Participants were right-handed according to self-report. Because the main goal of this experiment was to investigate effects of gender and autistic traits on social reward processing in healthy participants, participants of fMRI experiment 2 were stratified by the Autism Quotient (AQ; Baron-Cohen et al., 2002). History of neurological or psychiatric illness or use of any centrally acting drugs were contraindications for participation.

### Experimental set-up and data analyses

All experiments were programmed and conducted by means of the Presentation® software (version 0.71, Neurobehavioral Systems Inc., http://www.neurobs.com/).

### Behavioral experiments

#### German version of the verbal learning and memory test

All subjects of the first and second cohort performed a computer-based version of the VLMT, adapted with slight modifications from (Helmstaedter et al., 2001). In the second cohort, the full version was employed. After a list of 15 words had been presented on a computer screen, participants were asked to write down all the words they could remember. This procedure was repeated five times with the same list. Subsequently, an interference list consisting of 15 different words was presented once, and participants were instructed to write down as many words from the interference word list as they remember. An unexpected free-recall test of the original word list was conducted immediately after the recall of the distracter list and 30 min and 24 h after the last presentation of the original word list. In the immediate and 30 min free recall tests, subjects were instructed to write down the previously learned words. For the 24-h free recall test participants were unexpectedly contacted by email or telephone. In the first cohort a shortened version of the VLMT was used. This version consisted of three learning trials and an unexpected free-recall test 24 h after the last presentation of the original word list. The number of correctly recalled words was the dependent variable of interest.

#### Wechsler memory scale (WMS), logical memory subscale

All subjects of the first and second cohort took part in a computer-based German auditory version of the WMS for logical memory. The testing procedure had been adapted and slightly modified from (Härting et al., 2000). This subtest for logical memory of the WMS includes two short stories that were presented auditorily via headphones. After one story had been finished, subjects were asked to reproduce the story immediately in written form as precisely as possible. 30 min and 24 h after listening to the stories, delayed recall tests were performed. One day later, the 24-h recall test was conducted via email or by telephone. The number of correctly recalled *a priori* defined details of the stories was the relevant output, with a maximum of 25 items for each story. The number of recalled items was determined from the subjects' responses by two independent raters, and inter-rater reliability was calculated by computing Type 3 intra-class correlations (ICCs). All ICCs were *r* > 0.99. For data analysis purposes, the scores of both stories A and B were summed up for each condition (immediate recall, recall after 30 min, and recall after 24 h). In the first cohort, a shortened version of the logical memory test was used that consisted of immediate recall of story A and an unexpected free-recall test after 24 h. As in the second cohort, two independent raters assessed recall accuracy, and all Type 3 ICCs were significant (all *r* > 0.67).

#### Data analysis

To examine the influence of *RASGRF1* rs8027411 on memory performance in both experiments, we initially performed separate two-way analyses of covariance (ANCOVAs) for repeated measures with memory (*first cohort*: VLMT: number of correctly recalled items in trials 1–3 and 24 h delayed recall; WMS: number of correctly recalled items in immediate recall and 24 h delayed recall; second *cohort*: VLMT: number of correctly recalled items in trials 1–5, 5 min, 30 min and 24 h delayed recall; WMS: number of correctly recalled items in immediate recall, 30 min and 24 h delayed recall) as within-subject factor, *RASGRF1* genotype as between-subject factor, and age and gender as covariates of no interest. Because the VLMT results exhibited considerable ceiling effects, further ANCOVAs were computed with performance group (top 25%, medium 50%, bottom 25%) as an additional between-subjects factor (see Results section for details) Degrees of freedom were corrected for non-sphericity using the Greenhouse-Geisser correction. To assess directionality of genotype effects revealed by the ANCOVAs, we computed Spearman's non-parametric correlation analyses between the number of T alleles (0, 1, or 2) and memory performance. In the second cohort, we conducted the same correlation analyses as in the first cohort, but using a one-tailed threshold of *p* < 0.05, given our unidirectional hypotheses derived from the results of the first cohort.

#### Analysis controlling for ceiling effects in VLMT

Due to the observed ceiling effects (see Results section, Trial 3 in both cohorts) particularly in the second cohort, both cohorts were divided into three groups, based on the total value of memory performance (sum value of all trials). The upper quartile formed the group of *high performers*. The group of *low performers* comprised the lower quartile. The group of *moderate performers* refers to the area between the upper and lower quartiles. The statistical analyses described above, ANCOVA for repeated measures and Spearman correlations, were performed in consideration of the performance groups.

### Functional MRI experiment 1

#### Experimental paradigm (Duzel et al., 2011)

During the fMRI session the subjects performed an incidental encoding task. Eighty-eight novel images (44 indoor and 44 outdoor) and 22 repetitions of each of two familiar images (one indoor and one outdoor scene) were presented in a pseudo-randomized order. The two familiar images (“master pictures”) had been familiarized over five repetitions each directly before MRI acquisition. During the actual experiment, stimuli were shown for 2.5 s, each with an average inter-stimulus interval (ISI) of 1000 ms (jittered around the average ISI with an SD of 600 ms), during which a fixation cross was shown. The order of stimulus presentation was optimized for event-related fMRI time series sampling (Hinrichs et al., 2000). Participants indicated via button press whether an indoor or outdoor scene was presented. Supplementary Figure S1 depicts the structure of an example trial.

Approximately 90 min after the start of the fMRI session, participants performed a recognition memory task with a five-step confidence rating during which the 90 images from the fMRI session were presented randomly intermixed with 44 distractors that had not been presented before (22 indoor–22 outdoor). The task was performed outside the MR tomograph. Subjects rated their recognition confidence on a scale ranging from 1 to 5 (“1”: definitely new; “2”: likely new; “3”: unsure; “4”: likely old; “5”: definitely old). Based on these confidence ratings fMRI data were modeled.

#### MRI data acquisition

MRI data were acquired using a Verio Syngo 3T MR system (Siemens Medical Systems, Erlangen) with a 32-channel head coil. Prior to the functional MRI session, a T1-weigthed 3D Magnetization Prepared Rapid Acquisition Gradient Echo (MPRAGE) image was acquired (192 sagittal slices, image matrix = 256 × 256, field of view (FOV) = 256 × 256 mm^2^, slice thickness = 1 mm, *TE* = 4.37 ms, *TR* = 2500 ms, flip angle = 7°, voxel size of 1 × 1 × 1 mm^3^), followed by a single BOLD fMRI run during which our experiment took place. A total of 206 T2^*^-weighted gradient-echo echo-planar images (GE-EPIs) were acquired in a single session that lasted approximately 8 min (40 axial slices covering the entire brain; image matrix = 104 × 104; FoV = 208 × 208 mm; voxel size: 2 × 2 × 3 mm^3^; *TR* = 2400 ms; *TE* = 30 ms; flip angle 80°; odd-even interleaved acquisition order). Thereafter, a co-planar inversion recovery echo-planer image (IR-EPI, voxel size = 2 × 2 × 2 mm^3^) was acquired for spatial normalization (see below).

#### Data processing and analysis

Data were analyzed using Statistical Parametric Mapping (SPM8, Wellcome Department of Imaging Neuroscience, Institute of Neurology, London, UK). EPIs were corrected for acquisition delay and head motion. EPIs were co-registered to the mean image obtained from motion correction and used to determine normalization parameters for spatial normalization to the Montreal Neurological Institute (MNI) stereotactic coordinate system (voxel size = 2 × 2 × 2 mm^3^). Data were smoothed using an isotropic Gaussian kernel of 6 mm full width at half maximum.

Statistical analysis was performed using a two-stage mixed effects model. At the first stage, the stimulus-specific BOLD responses were modeled by convolving a delta function at stimulus onset with a canonical hemodynamic response function (HRF; Friston et al., 1998). The resulting time courses were resampled to the resolution of 1/TR (one time point for each scan) to form covariates of a general linear model (GLM). The GLM included separate regressors for each condition of interest (novel pictures, sorted by later recognition confidence, master pictures), the six rigid-body movement parameters determined from motion correction as covariates of no interest, and a single constant representing the mean over scans. Model estimation was performed using a restricted maximum likelihood (ReML) fit. At the second stage of the model, linear contrast images were computed for the novelty encoding condition (novel stimuli with recognition confidence ratings 4 and 5 compared to the master stimuli) and submitted to group-level random-effects analysis. To test for effects of *RASGRF1* rs8027411, a one-way ANCOVA was conducted with *RASGRF1* genotype as fixed factor, and age and sex as covariates of no interest. Because of our *a priori* hypotheses regarding the bilateral hippocampi, alpha error probabilities were adjusted for the volumes of these regions of interest (ROIs). The significance threshold was set to *p* < 0.05, family-wise error (FWE)-corrected for multiple comparisons. The ROIs of the left and right hippocampus (CA regions) were obtained from a probabilistic cytoarchitectonic atlas (SPM Anatomy Toolbox; Eickhoff et al., 2005).

### Functional MRI experiment 2

#### Experimental paradigm

After being positioned in the MR scanner, participants were given a short demonstration of the task and completed a practice session lasting 2:54 min (20 trials). This practice session should minimize learning effects during functional data acquisition and was intended to lead to a switching of reward responses from the moment of reward receipt to the time of reward anticipation (Wittmann et al., 2005; Krebs et al., 2009). After the practice session, the MR experiment was conducted. Once the first experimental session was completed, participants read the instruction of the second part of the task and completed another practice session lasting 2:54 min (20 trials).

Each of the two reward sessions consisted of 100 trials lasting 14:06 min (50 reward and 50 no-reward trials). A modified version of the monetary incentive delay task described previously (Wittmann et al., 2005; Krebs et al., 2009) was conducted in one of the two sessions. In the other session, the participant had the opportunity to get a social feedback instead of the monetary reward. The picture of a smiling face upon correct response represented this social feedback. The order of the runs (monetary vs. social) was counterbalanced across participants. Each trial started with the presentation of a cue picture for 1000 ms. Cues signaled either a potential reward or a neutral outcome. Participants were asked to pay attention to the cues in order to be aware of the reward status and to respond via button press (right index or middle finger) indicating whether they expected a reward or not. After a variable interval (delay, 500–3500 ms), a number comparison task (target, 250 ms; Pappata et al., 2002; Wittmann et al., 2005) followed. Participants were requested to give a speeded response whether a target number was larger or smaller than 5. The response deadline was adjusted individually based on reaction times in the immediately preceding session to attain a correct response rate of ~80%. During reward trials, participants could win 1€ (picture of 1€ coins) or positive social feedback (picture of smiling person) upon successful response in the number comparison task. In case of a lost trial (wrong or/and slow response), a black/white pattern noise-image was presented. During neutral trials, a black/white pattern noise-image was shown irrespective of outcome. A visual feedback (750 ms) was given 500–2500 ms after the presentation of the target and was followed by a variable fixation phase (1000–4000 ms). Supplementary Figure S2 shows the timing of a single trial.

Approximately 24 h after the start of the fMRI session, participants performed a recognition memory task with a five-step confidence rating during which the 200 cue images from the fMRI session were presented randomly intermixed with 100 distracters that had not been presented before. The task was performed outside the MRI scanner. Subjects rated their recognition confidence on a scale ranging from 1 to 5 (“5”: definitely new; “4”: likely new; “3”: unsure; “2”: likely old; “1”: definitely old). These confidence ratings were used to model the relationship between individual behavior and brain response during first-level fMRI data analysis. See Supporting Information for details on used reward stimuli and cues signaling reward.

#### MRI data acquisition

fMRI was performed using a 3 Tesla Siemens Magnetom Trio Scanner (SIEMENS Medical Systems, Erlangen, Germany) and a 12-channel phased array head coil. We collected structural (T1-weighted MPRAGE: 256 × 256 matrix; FOV = 256 mm; 96 2-mm sagittal slices) and functional images (Gradient-Echo-EPI-sequence; *TR* = 2000 ms; *TE* = 30 ms; FOV = 240 mm; flip-angle = 90°; matrix = 96 × 96; slice-thickness = 3 mm; 34 oblique slices parallel to the anterior commissure—posterior commissure voxel size = 2.5 × 2.5 × 3 mm^3^; two runs of 420 volumes).

#### Data processing and analysis

Image processing and statistical analyses were performed using SPM8 (http://www.fil.ion.ucl.ac.uk/spm/). EPIs were corrected for acquisition delay and head motion, spatially normalized and smoothed (isotropic Gaussian Kernel; FWHM = 8 mm). A high pass filter with a cut of frequency of 128 s was applied to the data. Statistical analysis was carried out using a two-stage mixed effects model. At the first stage, encoding processing-related hemodynamic responses were analyzed as a function of subsequent recognition memory success in the recognition memory test by creating separate regressors for each of the five confidence rates and reward categories. Thus, the final model contained two sessions (monetary/social) with 10 regressors of interest each (confidence level 1… 5 × reward/neutral). This model as fitted to the spatially preprocessed and temporally filtered data using a ReML fit, with movement parameters as covariates of no interest. Linear DM effect contrast images were computed (stimuli with recognition confidence ratings 1 and 2 compared to stimuli with recognition confidence ratings 4 and 5) and submitted to a second-level full-factorial random effects model with *RASGRF1* as fixed factor, and age and sex as covariates of no interest. As in Experiments 1, genotype-related effects were investigated with focus on the hippocampus, (*p* < 0.05, small-volume FWE-corrected), using the same ROIs as in the first experiment.

## Results

### Genotyping and demographics

Among the 355 study participants in the first cohort, we identified 95 individuals homozygous for the T allele of rs8027411, 186 heterozygotes, and 74 G homozygotes. The genotype distribution was in Hardy-Weinberg equilibrium (HWE) (χ^2^ = 0.94, *p* > 0.05). In the second cohort, the sample of 719 volunteers included 193 T homozygotes, 334 heterozygotes, and 192 G homozygotes. As in the first cohort, there was no significant deviation from HWE (χ^2^ = 3.62, *p* > 0.05). The *RASGRF1* genotypes TT, TG and GG did not differ in gender distribution, mean age or in the ratio of ametropia (for detailed demographic information see Table 1 and Supplementary Figure S3; information regarding ametropia is available only in the second cohort). However, direct comparison of individuals with myopia only and individuals with no refractive error revealed a trend for imbalanced distribution of myopia between the two homozygous groups (χ^2^ = 2.99, *p* = 0.084), with a higher prevalence among T homozygotes as reported previously (Hysi et al., 2010; see Supplementary Figure S3).

**Table 1:** **Demographic data**.

	**TT**	**TG**	**GG**	
**1ST COHORT: BEHAVIORAL EXPERIMENT**				
Women/Men	53/42	96/90	39/35	χ^2^ = 0.44; *p* = 0.801
Mean age	23.0 ± 3.1	23.1 ± 2.9	22.4 ± 2.7	*F*_(2, 352)_ = 1.55; *p* = 0.213
**2ND COHORT: BEHAVIORAL EXPERIMENT**				
Women/Men	110/83	175/159	90/102	χ^2^ = 3.96; *p* = 0.138
Mean age	23.8 ± 2.9	23.9 ± 2.6	23.5 ± 2.9	*F*_(2, 716)_ = 0.93; *p* = 0.394
Ametropia/no ametropia	89/74	151/136	76/90	χ^2^ = 2.93; *p* = 0.231
**2ND COHORT: ENCODING OF NOVEL SCENES (fMRI EXPERIMENT)**				
Women/Men	16/15	30/24	12/22	χ^2^ = 3.57; *p* = 0.168
Mean age	24.0 ± 2.7	24.4 ± 2.6	24.6 ± 2.5	*F*_(2, 116)_ = 0.38; *p* = 0.684
**2ND COHORT: REWARD-DEPENDENT MEMORY ENCODING (fMRI EXPERIMENT)**				
Women/Men	3/9	19/12	9/11	χ^2^ = 4.77; *p* = 0.092
Mean age	24.9 ± 1.9	24.0 ± 2.4	25.2 ± 3.6	*F*_(2, 60)_ = 1.34; *p* = 0.269

*Gender distribution and age (average ± SD) of the participants, separated by genotypes (TT, TG, GG). For the 2nd cohort, occurrence of ametropia was also estimated*.

### Effects of *RASGRF1* rs8027411 on memory performance

**Figure 1:**
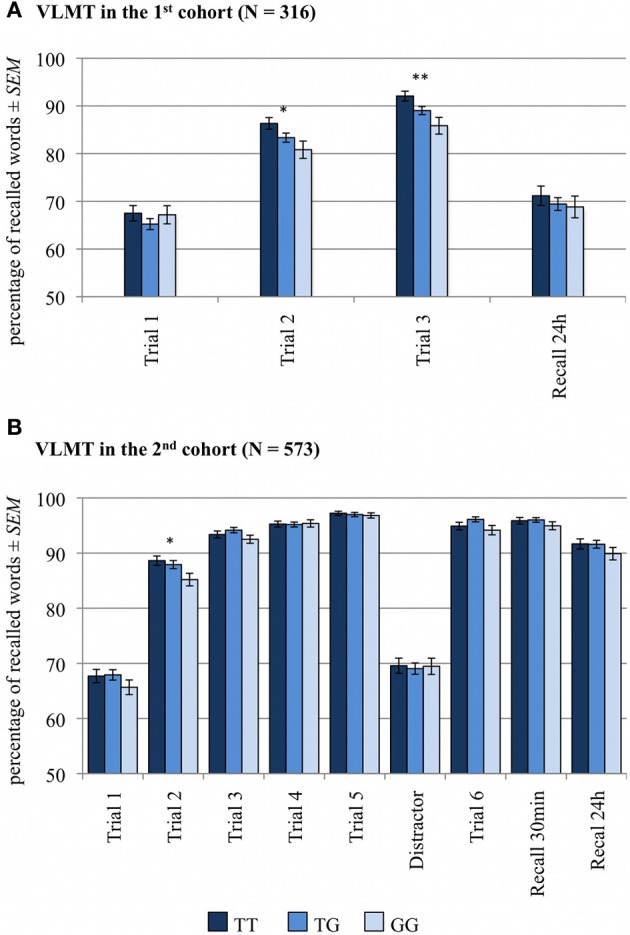
**Memory effect of *RASGRF1* in Verbal Learning and Memory (VLMT)**. Higher % values indicate better memory performance. **(A)** In the first cohort we observed a significant positive correlation between T allele count and recall performance in the second and third learning trials. **(B)** In the second cohort we found a positive correlation between the T allele count and recall performance in the second learning trial. ^*^Significant at *p* < 0.05; ^**^Significant at *p* < 0.01.

**Figure 2:**
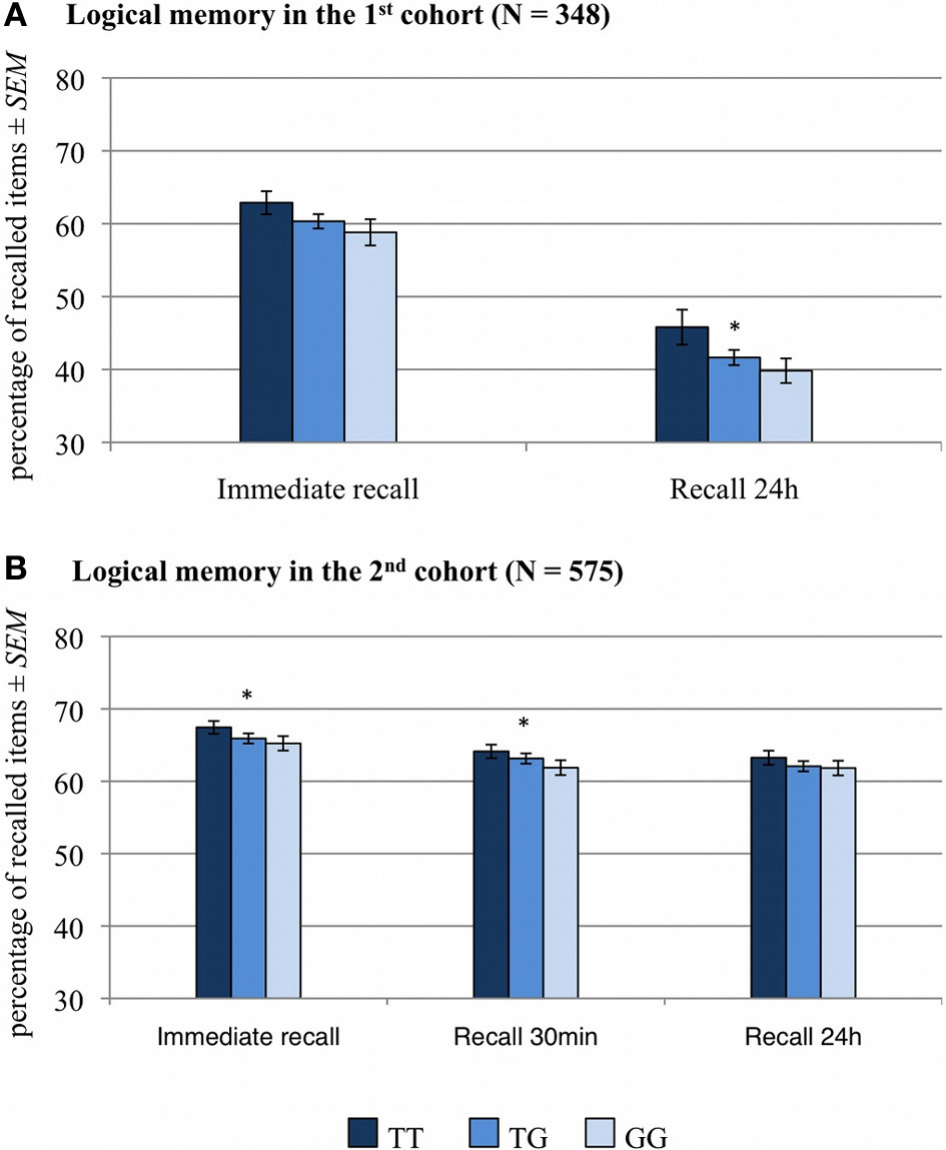
**Memory effect of *RASGRF1* in a logical memory task WMS)**. Higher % values indicate better memory performance. **(A)** In the first cohort correlation analyses revealed a significant correlation between the T allele count and performance in the 24-h delayed recall test and a trend-wise positive correlation between T allele count and immediate recall performance. **(B)** In the second cohort, T allele count was positively correlated with performance in the immediate recall test and in delayed recall after 30 min. ^*^Significant at *p* < 0.05.

The numbers of correctly recalled words (VLMT) and correctly recalled story items (WMS, logical memory) are displayed in Figures 1, 2 and Table 2, separated by *RASGRF1* genotype.

**Table 2:** **Statistics of the behavioral data**.

	**No. of correctly recollected items**		
	**TT**	**TG**	**GG**
**VERBAL LEARNING AND MEMORY TEST**			
**1st cohort**			
Trial 1	10.1 ± 2.2	9.8 ± 2.3	10.1 ± 2.3
Trial 2	13.0 ± 1.6	12.5 ± 1.9	12.1 ± 2.2
Trial 3	13.8 ± 1.4	13.4 ± 1.7	12.9 ± 2.1
Recall 24 h	10.7 ± 2.7	10.4 ± 2.6	10.3 ± 2.8
**2nd cohort**			
Trial 1	10.2 ± 2.3	10.2 ± 2.3	9.9 ± 2.5
Trial 2	13.3 ± 1.6	13.2 ± 1.8	12.8 ± 2.1
Trial 3	14.0 ± 1.2	14.1 ± 1.2	13.9 ± 1.4
Trial 4	14.3 ± 1.0	14.3 ± 1.1	14.3 ± 1.2
Trial 5	14.6 ± 0.7	14.6 ± 0.9	14.5 ± 0.9
Distractor trial	10.4 ± 2.6	10.4 ± 2.5	10.4 ± 2.7
Trial 6	14.2 ± 1.3	14.4 ± 1.1	14.1 ± 1.6
Recall 30 min	14.4 ± 1.1	14.4 ± 1.0	14.2 ± 1.3
Recall 24 h	13.8 ± 1.8	13.7 ± 1.7	13.5 ± 2.1
**LOGICAL MEMORY**			
**1st cohort**			
Immediate recall	15.7 ± 3.8	15.1 ± 3.3	14.7 ± 3.8
Recall 24 h	11.5 ± 5.8	10.4 ± 3.5	10.0 ± 3.6
**2nd cohort**			
Immediate recall	33.7 ± 5.7	33.0 ± 5.6	32.6 ± 6.1
Recall 30 min	32.1 ± 5.9	31.6 ± 5.7	30.9 ± 6.3
Recall 24 h	31.6 ± 6.3	31.0 ± 5.7	30.9 ± 6.3

*Means and standard deviations (SD) are reported, separated by genotype (TT, TG, GG). Verbal Learning and Memory Test: minimum score 0, maximum score 15. Logical memory: minimum score 0, maximum score 25 in the 1st cohort and 50 in the 2nd cohort)*.

#### Verbal learning and memory test (VLMT)

A two-way ANCOVA for repeated measures with memory (number of correctly recalled items in trials 1–3 and in 24-h free recall trial) as within-subject factor, *RASGRF1* genotype as between-subject factor and age and gender as covariates of no interest revealed a significant main effect of memory [*F*_(3, 933)_ = 9.5; *p* < 0.001], reflecting the learning curve from the first to third trial, and a significant memory by *RASGRF1* genotype interaction [*F*_(6, 933)_ = 2.2; *p* = 0.050], albeit there was no significant main effect for *RASGRF1* genotype [*F*_(2, 311)_ = 1.4; *p* = 0.247]. An overview of significant effects of considered covariates of no interest is given in Supplementary Table S2. To assess directionality of the observed interaction, we computed a non-parametric correlation analysis between the number of *RASGRF1* T alleles (GG = 0, TG = 1, TT = 2) and memory performance or number of correctly recalled words, respectively. There was a significant positive correlation between T allele count and recall performance in the second (Spearman's ρ = 0.13, *p* = 0.022) and third (Spearman's ρ = 0.15, *p* = 0.008) learning trials, but no correlation between *RASGRF1* and the initial memory performance in the first learning trial as well as in the free recall test after 24 h. In the second cohort, partially replicating our previous results, we observed a positive correlation between the T allele count and recall performance in the second learning trial (Spearman's ρ = 0.07, *p* = 0.043 one-tailed). Putatively due to ceiling effects resulting from generally higher performance, no further significant correlations were observed in the second cohort (see Supplementary Table S3). The ANCOVA for repeated measures revealed a significant effect for the factor memory in the second cohort [*F*_(7, 3976)_ = 12.14; *p* < 0.001], but no genotype-associated effects (*p* > 0.123). Significant effects of considered covariates of no interest are summarized in Supplementary Table S2.

#### VLMT results controlled for ceiling effects

A detailed overview of all significant effects is given in Supplementary Table S4. The ANCOVA for repeated measures with memory (*first cohort*: number of correctly recalled items in trials 1–3 and 24 h delayed recall; second *cohort*: number of correctly recalled items in trials 1–5, 5 min, 30 min and 24 h delayed recall) as within-subject factor, *RASGRF1* genotype and performance group as between-subject factors, plus age and gender as covariates of no interest revealed main effects of genotype in both cohorts [first cohort: *F*_(2, 305)_ = 3.08; *p* = 0.048; second cohort: *F*_(2, 562)_= 4.17; *p* = 0.016]. In addition, we observed a significant genotype by performance group interaction in the second cohort [*F*_(4, 562)_ = 4.87; *p* = 0.001], and significant three-way interactions (memory by genotype by performance group) in both cohorts [first cohort: *F*_(12, 915)_ = 1.91; *p* = 0.039; second cohort: *F*_(28, 3934)_ = 1.69; *p* = 0.036]. Furthermore, a significant memory by genotype interaction was observed in the first cohort [*F*_(6, 915)_ = 3.20; *p* = 0.007], while the latter interaction showed only a trend toward significance level in the second cohort [*F*_(14, 3934)_ = 1.83; *p* = 0.060]. To further assess whether the observed interactions were indeed related to ceiling effects, we performed the same ANCOVAs separately for each performer group. In the group of *low performers* we observed a main effect of genotype in both cohorts [first cohort: *F*_(2, 75)_ = 3.38; *p* = 0.039; second cohort: *F*_(2, 156)_ = 4.24; *p* = 0.016]. Moreover, in the first cohort, we detected a significant memory genotype interaction in the group of *low performers* [*F*_(6, 225)_ = 3.11; *p* = 0.009]. In both cohorts there were no significant genotype-related effects in the group of *moderate* and *high performers* (*p* > 0.073). *Post-hoc* non-parametric correlation analysis between the number of T alleles and memory performance were therefore limited to the group of *low performers*. In the first cohort, we observed positive correlations between T allele count and memory performance in the second (Spearman's ρ = 0.30, *p* = 0.007) and in the third learning trial (Spearman's ρ = 0.35, *p* = 0.001). In the second cohort, the number of the T alleles was positively correlated with performance in the second learning trial (Spearman's ρ = 0.28, *p* < 0.001 one-tailed).

#### Wechsler memory scale (WMS), logical memory

A two-way ANCOVA for repeated measures with memory performance (number of correctly recalled items in immediate recall and 24 h delayed recall) as within-subject factor, *RASGRF1* genotype as between-subject factor, and age and gender as covariates of no interest revealed a significant main effect of *RASGRF1* genotype [*F*_(2, 343)_ = 3.0; *p* = 0.050]. A short summary of significant effects of considered covariates of no interest is displayed in Supplementary Table S2. *Post-hoc* correlation analyses showed a significant correlation between the T allele count and performance in the 24-h delayed recall test (Spearman's ρ = 0.11, *p* = 0.042). There was also a trend for a positive correlation between T allele count and immediate recall performance (Spearman's ρ = 0.10, *p* = 0.058, see Supplementary Table S3). To verify robustness of the finding observed in the first cohort, we computed Spearman's correlations between T allele count and performance in logical memory scale of the WMS in the second cohort. As in the first cohort, the T allele was associated with better memory performance. Specifically, T allele count was positively correlated with performance in the immediate recall test (Spearman's ρ = 0.07, *p* = 0.050 one-tailed) and in delayed recall after 30 min (Spearman's ρ = 0.08, *p* = 0.033 one-tailed). There was no significant correlation between the T allele count and delayed recall after 24 h (see Supplementary Table S3). In the second cohort, the performed ANCOVA for repeated measures showed no genotype-related effects on memory performance in logical memory (*p* > 0.184). The significant effects of considered covariates of no interest are displayed in Supplementary Table S2.

### Effects of *RASGRF1* rs8027411 on hippocampus-dependent novelty processing

To assess the effects of *RASGRF1* rs8027411 on hippocampal function during memory encoding, we investigated the effect of T allele count on the hippocampal BOLD response to novel stimuli using a well-established rapid fMRI paradigm during which novel scenes are presented randomly intermixed with highly familiar “master” pictures and encoded incidentally (Duzel et al., 2011).

#### Behavioral data

The reaction times (RTs) of the indoor/outdoor decision during the encoding phase in the MRI scanner were comparable across the *RASGRF1* genotype groups [study participants were not instructed to react as fast as possible; see Supplementary Table S5 for the RTs; One-Way ANOVA *F*_(2, 116)_ = 1.68; *p* = 0.191]. Error rates were very low in all three groups (below 1%) and did not differ significantly as a function of *RASGRF1* genotype [One-Way ANOVA *F*_(2, 116)_ = 0.67; *p* = 0.512]. In the recognition phase all confidence ratings 1–5 were recorded and analyzed to investigate a genotype-dependent effect on memory performance (see Supplementary Table S5 for detailed information regarding recognition performance). A one-way analysis of variance (ANCOVA) with the allele groups as within subject-factor, the corrected hit rate as dependent variable, and age and gender as covariates of no interest revealed no significant effect of the *RASGRF1* genotype [*F*_(2, 114)_ = 0.39; *p* = 0.680]. When computing the receiver operating characteristic (ROC) of recognition confidence responses, we also observed no significant effect of *RASGRF1* genotype on the accuracy parameter d' [One-Way ANCOVA; *F*_(2, 114)_ = 0.16; *p* = 0.851].

#### Functional MRI data

**Figure 3:**
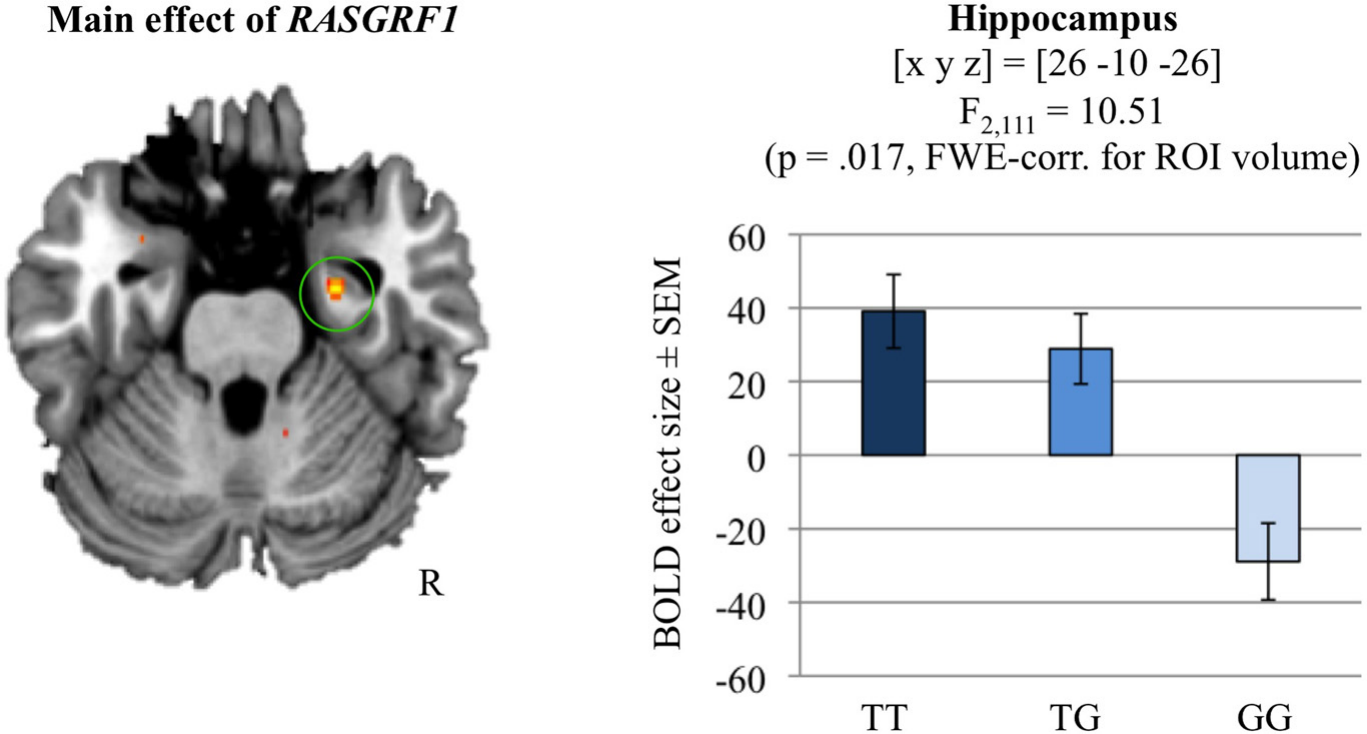
**Hippocampal novelty processing and its modulation by *RASGRF1* genotype (main effect of * RASGRF1*)**. TT carriers exhibited increased hippocampal activation, when compared to G allele carriers. This effect was significant at *p* < 0.017, FWE-corrected for ROI volume. Activations are superimposed on the MNI template brain provided by MRIcron. Coordinates are in MNI space. Bar plots depict contrasts of parameter estimates at the peak coordinate separated by genotype. Error bars depict standard errors of the mean.

Independently of genotype, the analysis of the novelty contrast (BOLD responses to novel stimuli that were later correctly recognized compared to the highly familiar “master” pictures) revealed significant activations in a temporo-occipital network including the right hippocampus [(x, y, z) = (28, −42, −12); *t*_(111)_ = 26.59; *p* < 0.001, whole-brain FWE-corrected; for further novelty-related activations see Supplementary Table S6]. An ANCOVA model with *RASGRF1* as fixed factor and age and gender as covariates of no interest revealed a significant main effect of genotype in the right hippocampus [(x, y, z) = (26, −10, −26); *F*_(2, 111)_ = 10.51; *p* = 0.017, FWE-corrected for hippocampus ROI; ω^2^ = 0.138]. Specifically, T allele homozygotes showed the most pronounced activation of the right hippocampus during novelty processing (Figure 3). Supplementary Table S7 displays a comprehensive list of further *RASGRF1* genotype-associated brain activation differences during encoding of novel scenes, but none of those was significant after whole-brain FWE correction.

### Effects of *RASGRF1* rs8027411 on reward-dependent memory encoding

To further substantiate the role of the *RASGRF1* polymorphism as a genetic modifier of hippocampus-dependent memory, we performed *post-hoc* analyses in a previously acquired reward-dependent memory task. The task was primarily designed to investigate the effects of monetary and social reward on memory encoding processes as a function of gender and autistic traits in young, healthy individuals and was based on previous paradigms of rewardmotivated encoding (Wittmann et al., 2005; Krebs et al., 2009).

#### Behavioral data

We performed two ANCOVAs for repeated measures (separately for each reward type) with corrected hit rate for reward predicting cues and corrected hit rate for no reward predicting cues as within-subject factor, *RASGRF1* genotype as between-subject factor, and age and gender as covariates of no interest. In the monetary reward condition, we observed a significant main effect of genotype [*F*_(2, 58)_ = 3.83; *p* = 0.027]. In the social reward condition only a trend toward main effect of reward was observed [*F*_(1, 58)_ = 3.93; *p* = 0.052], but no genotype-related effects (*p* > 0.507). Based on the significant main effect of *RASGRF1* genotype on the rate of correct responses in the monetary reward condition only, subsequent analyses were focused on the monetary condition. In line with our behavioral and neuroimaging results reported above (VLMT, WMS logical memory) and the results from the fMRI paradigm (encoding of novel scenes), T allele homozygotes exhibited better recognition memory performance for pictures predicting monetary reward [two-sample *t-test*: *t*_(61)_ = 2.02; *p* = 0.024, one-tailed; see Supplementary Table S5]. These results did not change significantly when AQ was included as a covariate.

#### Functional MRI data

**Figure 4:**
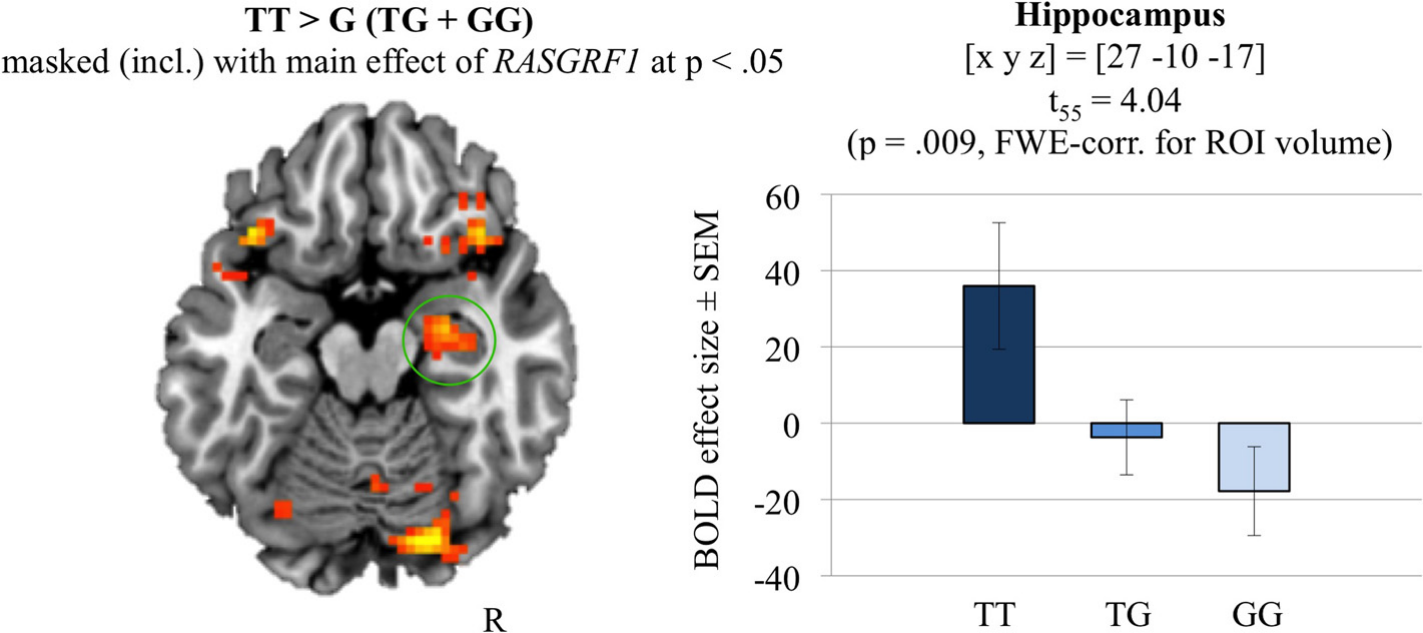
**The hippocampal recognition-encoding response for monetary reward-predicting items and its modulation by *RASGRF1* genotype [constrast TT vs. G (GT+GG) masked (incl.) with the main effect of *RASGRF1* at *p* < 0.05]**. TT carriers showed highest hippocampal activation, when compared to G allele carries (*p* = 0.009, FWE-corrected for ROI volume). Activations are superimposed on the MNI template brain provided by MRIcron. Coordinates are in MNI space. Bar plots depict contrasts of parameter estimates at the peak coordinate separated by genotype. Error bars depict standard errors of the mean.

Group level analysis of the effect of *RASGRF1* genotype followed the difference due to memory procedure (DM; for a review, see Paller and Wagner, 2002), i.e., brain responses to subsequently remembered reward predicting items were compared to brain responses to rewarded items that were later forgotten (Wittmann et al., 2005). Irrespective of genotype, successful memory encoding was associated with activation of the right hippocampus [(x, y, z) = (30, −19, −17); *t*_(55)_ = 4.62; *p* = 0.002, FWE-corrected for hippocampus ROI volume]. The two-sample *t*-test comparing the monetary reward DM contrasts of T allele homozygotes to those of G allele carriers (inclusively masked with the main effect of *RASGRF1*, thresholded at *p* < 0.05) revealed a significantly higher right hippocampal activation in the T homozygotes compared to G carriers [(x, y, z) = (27, −10, −17); *t*_(55)_ = 4.04; *p* = 0.009, FWE-corrected for hippocampal ROI volume; ω^2^ = 0.198; see Figure 4]. Further genotype-related activation differences that survived whole-brain FWE correction are reported in Supplementary Table S8. Including AQ as a further covariate did not significantly affect the fMRI results regarding *RASGRF1* genotype.

## Discussion

In our two independent study cohorts of young, healthy participants (*n*_1_ > 316, *n*_2_ > 573), we observed an association of the SNP rs8027411, located in the transcription initiation region of the *RASGRF1* gene, with memory performance in hippocampus-dependent tasks. Specifically, individuals carrying the T allele, and particularly T homozygotes, showed better memory performance in the early encoding phases of the VLMT and the WMS, logical memory section, than homozygous G allele carriers. In line with these findings, T homozygous individuals exhibited increased hippocampal activation during encoding of novel scenes and, as a trend, during encoding of pictures associated with monetary reward.

### *RASGRF1* modulates human memory performance and hippocampus function

We observed a relationship between a genetic variation in the *RASGRF1* transcription initiation region (rs8027411) with human learning and memory processes in young, healthy subjects. Specifically, the individual T allele count (0, 1, 2) was significantly correlated with memory performance in early encoding phases of the learning tasks, suggesting a dosage effect of the T allele on human memory function, possibly mirroring the gene-dose effect of *RASGRF1* expression in rodents (Fasano et al., 2009). The association was observed in two independent cohorts, and we therefore conclude that the effects of *RASGRF1* are unlikely to result from spurious associations within a specific cohort. In the VLMT, particularly in the second cohort, effects of rs8027411 on memory performance were primarily observed during early stages of learning. Because participants were all young, healthy students tested under optimal conditions, it is plausible that this results in a ceiling effect, with participants having reached their maximum performance after the third learning trial. The ceiling effects analyses revealed that the found *RASGRF1*-related effects on memory performance are limited to the group of low performers. These findings provide an additional insight into the results on the overall performance level. They show that *RASGRF1* genotype-related differences in memory performance were, at least in case of the VLMT, most pronounced in the group of low performers. In the WMS logical memory scale, on the other hand, no ceiling effects were observed. The fact that all correlations between T allele count and memory performance were rather weak, with small effect sizes, seems plausible when considering the complex character of the studied traits. It should, however, be noted that a single common genetic variant would be unlikely to exert major influences on cognitive function. Moreover, in the relatively homogenous cohorts of high-functioning young adults, variability of cognitive performance is already smaller than in a population with a wider range in age and educational background, and any individual differences attributable to common genetic variations would be expected to be small. On the other hand, genotype-related differences within such homogenous cohorts are less likely to result from stratification effects.

In accordance with the positive relationship between T allele carrier status and memory performance we observed a modifying effect of *RASGRF1* genotype on hippocampal activity during encoding of novel scenes and of reward-associated stimuli. In both fMRI experiments, T allele homozygotes exhibited higher hippocampal BOLD responses when compared to G allele carriers. Genetically mediated differences in hippocampal activation during encoding have previously been linked to effects of the respective gene variants on memory function at a behavioral level, and carriers of the variants associated with better memory performance have typically shown stronger memory-related hippocampal activation (Hariri et al., 2003; Bertolino et al., 2006; Schott et al., 2011). In the present study, T homozygotes exhibited a behavioral advantage during memory task performance in VLMT, WMS logical memory, and in the reward-dependent memory encoding task in fMRI. This advantage was, however, not found in the task of the first fMRI experiment (encoding of novel scenes). We suggest that this may also be related to a ceiling effect since hit rate was invariably high across participants. It should be noted that differences in local BOLD signal changes have been suggested to be more closely related to the actual cellular effects mediated by genetic variations than behavioral between-group differences and might thus be detected in cohorts even without observable behavioral effects (Meyer-Lindenberg and Weinberger, 2006; Mier et al., 2010).

Our observation that a genetic variant in a putative *RASGRF1* regulatory region affects human memory performance and memory-related hippocampal activity is in line with findings from animal research that have previously implicated *RASGRF1* in learning and memory processes (Brambilla et al., 1997; Giese et al., 2001; d'Isa et al., 2011). Depending on the tested *RASGRF1* knockout mouse strain, different forms of memory were affected. Brambilla et al. (1997) primarily observed impaired amygdala-dependent memory (fear conditioning, inhibitory avoidance) in *RASGRF1* knockout mice compared to wild-type mice. Moreover, Giese et al. (2001) observed deficits in a hippocampus-dependent memory task (Morris water maze) in a different *RASGRF1*-deficient mouse strain.

In fMRI experiment 2, the behavioral and neural effect of *RASGRF1* rs8027411 genotype on memory function was reliably observed in the (monetary) reward condition only. Interestingly, in mice a clear gene-dosage effect of *RASGRF1* on reward sensitivity was reported (Fasano et al., 2009).

As a nucleotide exchange factor that activates Ras (Raaijmakers and Bos, 2009), *RASGRF1* is involved in stimulating the ERK signaling pathway, one of the pivotal molecular cascades implicated in the expression of LTP and in the long-term consolidation of memory traces, a process that engages a distributed network of brain regions, including the neocortex, the hippocampus, the amygdala, and the striatum (Brambilla et al., 1997; Blum et al., 1999). Importantly, *RASGRF1* is expressed at high levels in hippocampus, neocortex, and thalamus (Brambilla et al., 1997). Within the hippocampus, *RASGRF1* deficiency also has impact on the expression of other genes involved in neural plasticity (Fernandez-Medarde et al., 2007). Further research, however, is required to elucidate the specific effects of rs8027411 on *RASGRF1* expression and function. The SNP is located 78 kb upstream of the human *RASGRF1* gene in a putative transcription initiation site (Hysi et al., 2010). The gene contains 28 exons, and at least three protein-coding isoforms are known, resulting from alternative splicing. An *in silico* analysis revealed several predicted transcription factor binding sites which are affected by the T/G SNP (see Supplementary Table S9). Hysi and colleagues pointed out that the variant rs8027411 resides in a 120 kb genomic region of high linkage disequilibrium (LD) that overlaps the *RASGRF1* transcription initiation site and part of the gene. It can thus not be excluded that the SNP might not be a causal variant but in LD with genetic variations that actually mediate the observed cognitive and visual phenotypes at systems level.

### *RASGRF1* genetic variant, refractive error, and human cognitive function

*RASGRF1* rs8027411 was first identified in a GWAS of myopia as a risk locus (Hysi et al., 2010). In that study, the T allele was associated with myopia, and Hysi and colleagues related their results to animal research showing that *RASGRF1*-deficient mice displayed retinal vision impairments such as photoreception deficits (Fernandez-Medarde et al., 2009) as well as larger crystalline lenses and, as a result, larger refractive power, when compared to wild-type mice (Hysi et al., 2010). In the present study, we also observed nominally higher prevalence of myopia in rs8027411 T homozygotes (as assessed by self-report; see Supplementary Figure S3). While this association did not reaching significance when employing more conservative testing, a statistical trend was observed. It should be noted that our cohort consisted of young, healthy participants with comparable educational level. In a larger and more heterogeneous cohort, the effect might be more pronounced (Hysi et al., 2010; Verhoeven et al., 2013).

The present data, along with the previously published GWAS, suggest that the same genetic variation, rs8027411 T, adversely affects vision, but exerts beneficial effects on memory performance. Therefore, rs8027411 may serve as an example that certain variants with apparently deleterious effects are present in the population if they are potentially advantageous in other biological processes. Given the small effect sizes, this interpretation remains, however, tentative. The relationship between refractive error and human cognitive function is complex. Myopia is overrepresented in individuals with high intelligence (for review see Czepita et al., 2008) as well as in humans with intellectual disability or mental retardation (Van Splunder et al., 2004; for review see Warburg, 2001), and developmental mechanisms have been suggested to underlie this phenomenon (Mak et al., 2006). In the present study, we investigated two cohorts of young, healthy adults with normal to above-average general intellectual ability. In a population thus selected, it is, in our view, not surprising that a genetic variant previously associated with myopia also predicted better memory performance. With the likely role of *RASGRF1* in regulating long-term plasticity mechanisms (see, e.g., Fernandez-Medarde et al., 2007), our results support the notion that molecular processes within the same biological pathway may underlie both developmental plasticity as well as adult neuronal plasticity.

## Conclusion

Our results provide evidence for a role of *RASGRF1* in human hippocampus-dependent memory function with carriers of the rs8027411 T allele exhibiting better memory performance and higher hippocampal activity. Moreover, with *RASGRF1* rs8027411 having been linked to myopia, our findings also provide an example for the same genetic variant exerting pleiotropic effects at systems level and thereby conferring both advantageous and disadvantageous effects on distinct complex phenotypes.

### Conflict of interest statement

The authors declare that the research was conducted in the absence of any commercial or financial relationships that could be construed as a potential conflict of interest.
