# Supplementary material for: Genetic variation of the RASGRF1 regulatory region affects human hippocampus-dependent memory
Source: Front Hum Neurosci. 2014 Apr 29;8:260. doi: 10.3389/fnhum.2014.00260 (PMC4010733; doi:10.3389/fnhum.2014.00260)
Supplement: Supplementary file 1 [file DataSheet1.PDF]

## Supplementary Online Information

## Supplementary Methods

### *Functional MRI experiment 2: Reward-related memory encoding*

*Reward stimuli:* The set of social reward stimuli consisted of 95 pictures of smiling women, men, children, and couples. Before actual data acquisition, stimuli were evaluated regarding sympathy and attraction by a group of 31 independent raters who did not participate in the main experiment (16 women, mean age ± SD: 26.5 ± 3.79 years); 15 men, mean age ± SD: 28 ± 3.88 years). A 5-point scale was used for rating of sympathy and attraction. Additionally, the Self-Assessment Manikin (Bradley and Lang, 1994), which enables the rating of the three dimensions valence, arousal and dominance, was used to further characterize the stimulus set. Supplementary Table S10 displays the behavioral data of the evaluation of the stimulus set. The set of monetary reward stimuli consisted of 15 images of 1€ coins and the set of neutral outcome stimuli contained five black/white pattern noise-pictures. Each picture had a size of 550 x 700 pixels.

*Cues signaling reward:* Cue pictures consisted of photographs of simple objects that belonged to six categories (vehicles, bags, furniture, music instruments, clothes, kitchen devices). Both types of reward were separated in two experimental sessions to minimize the number of cues to be memorized. For each participant and session (monetary vs. social), two categories were randomly chosen to signal a potential reward or neutral outcome, respectively.

## Supplementary Tables

**Table S1:** Genotyping.

| | ***RASGRF1*** **(**rs8027411) |
|---|---|
| Primer forward | *5'- GAG CCA GGT GCT TCA GGA GCT TAA GG -3'* |
| Primer reverse | *5'- GAT TGC TTG AGT CCA GGA AGT TGA GGC TG -3'* |
| annealing temperature | 61°C |
| Enzyme | MnlI (Fermentas) cuts G |
| agarose gel | 3,50% |
| fragment length | T-allele: 213 bp<br>G-allele: 25 + 188 bp |
| | 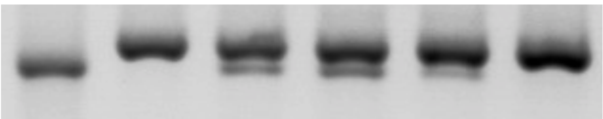<br>G/G T/T T/G T/G T/G T/T<br>25bp not visible under UV light, vanishes in primer cloud) |

Primers, annealing temperature, digestion enzyme and fragment length are shown.

**Table S2:** Significant effects of covariates of no interest

| | *F(p)* |
|---|---|
| **1st cohort** | |
| Verbal learning and memory test (N = 316) | |
| Main effect of gender | $F_{1,311}$ = 23.42 (<.001) |
| Logical memory (N = 348) | |
| Memory by age interaction | $F_{1,343}$ = 5.33 (.022) |
| **2nd cohort** | |
| Verbal learning and memory test (N = 573) | |
| Memory by gender interaction | $F_{14,3976}$ = 9.10 (<.001) |
| Main effect of gender | $F_{1,568}$ = 46.26 (<.001) |
| Main effect of age | $F_{1,568}$ = 6.72 (.010) |
| Logical memory (N = 575) | |
| Main effect of gender | $F_{1,570}$ = 17.53 (<.001) |
| Main effect of age | $F_{1,343}$ = 8.31 (.004) |

F-statistics and *p*- values are based on exploratory two-way ANCOVA for repeated measures with memory (*first cohort*: VLMT: number of correctly recalled items in trials 1 to 3 and 24h delayed recall; WMS: number of correctly recalled items in immediate recall and 24h delayed recall; *second cohort*: VLMT: number of correctly recalled items in trials 1 to 5, 5min, 30min and 24h delayed recall; WMS: number of correctly recalled items in immediate recall, 30min and 24h delayed recall) as within-subject factor, *RASGRF1* genotype as between-subject factor and age and gender as covariates of no interest performed in the 1st cohort.

**Table S3:** Detailed behavioral data (Spearman correlation)

| | | *r* | *p* |
|---|---|---|---|
| **Verbal learning and memory test** | | | |
| 1st cohort (N = 316) | | | |
| | Trial 1 | .02 | .726 |
| | Trial 2 | .13 | **.022*** |
| | Trial 3 | .15 | **.008**** |
| | Recall 24h | .05 | .389 |
| 2nd cohort (N = 573) | | | |
| | Trial 1 | .05 | .134 |
| | Trial 2 | .07 | **.043*** |
| | Trial 3 | .02 | .294 |
| | Trial 4 | -.04 | .148 |
| | Trial 5 | <-.01 | .492 |
| | Distractor trial | .01 | .370 |
| | Trial 6 | .01 | .409 |
| | Recall 30min | .02 | .318 |
| | Recall 24h | .04 | .197 |
| **Logical memory** | | | |
| 1st cohort (N = 348) | | | |
| | Immediate recall | .10 | .058 |
| | Recall 24h | .11 | **.042*** |
| 2nd cohort (N = 575) | | | |
| | Immediate recall | .07 | **.050*** |
| | Recall 30min | .08 | **.033*** |
| | Recall 24h | .05 | .097 |

Spearman correlations between *RASGRF1* polymorphism (T allele frequency) and memory performance across different trials. All *p*-values are two-tailed for the behavioral data of the 1st cohort for which we had no directed hypothesis. For the behavioral data of the 2nd cohort, all *p*-values are one-tailed. * significant at $p < .05$; ** significant at $p < .01$.

**Table S4:** Genotype related effects on the memory performance in VLMT

| | F-Test (p) |
|---|---|
| **First cohort** | |
| **overall** | |
| main effect of *RASGRF1* | $F_{2,305}$ = 3.08 (.048) |
| main effect of performance group | $F_{2,305}$ = 591.07 (<.001) |
| main effect of memory | $F_{3,915}$ = 9.80 (<.001) |
| memory x gender | $F_{3,915}$= 4.69 (.005) |
| *RASGRF1* x memory | $F_{6,915}$ = 3.20 (.007) |
| memory x performance group | $F_{6,915}$ = 20.07 (<.001) |
| *RASGRF1* x memory x performance group | $F_{12,915}$ = 1.91 (.039) |
| **high performers** | |
| main effect of memory | $F_{3,255}$ = 5.02 (.007) |
| memory x gender | $F_{3,255}$ = 3.39 (.035) |
| **moderate performers** | |
| Main effect of memory | $F_{1,423}$ = 5.67 (.001) |
| **low performers** | |
| main effect of *RASGRF1* | $F_{2,75}$ = 3.38 (.039) |
| *RASGRF1* x memory | $F_{6,225}$ = 3.11 (.009) |
| **Second cohort** | |
| **Overall** | |
| main effect of *RASGRF1* | $F_{2,562}$ = 4.17 (.016) |
| main effect of performance group | $F_{2,562}$ = 807.76 (<.001) |
| *RASGRF1* x performance group | $F_{4,562}$ = 4.87 (.001) |
| main effect of memory | $F_{7,3934}$ = 15.23 (<.001) |
| *RASGRF1* x memory | $F_{14,3934}$ = 1.83 (.060) |
| memory x performance group | $F_{14,3934}$ = 66.93 (<.001) |
| *RASGRF1* x memory x performance group | $F_{28,3934}$ = 1.69 (.036) |
| **moderate performers** | |
| main effect of memory | $F_{7,1701}$ = 9.30 (<.001) |
| **low performers** | |
| main effect of memory | $F_{7,1092}$ = 6.30 (<.001) |
| main effect of *RASGRF1* | $F_{2,156}$ = 4.26 (.016) |

The statistics are reported separately for both cohorts and performance groups. F-statistics and *p*- values are based on ANCOVA for repeated measures with memory (*first cohort*: number of correctly recalled items in trials 1 to 3 and 24h delayed recall; *second cohort*: number of correctly recalled items in trials 1 to 5, 5min, 30min and 24h delayed recall) as within-subject factor, *RASGRF1* genotype and performance group (in the overall analysis) as between-subject factor and age and gender as covariates of no interest.

**Table S5:** Descriptive statistics of the behavioral data

| | | | TT | TG | GG |
|---|---|---|---|---|---|
| **fMRI experiment 1: Encoding of novel scenes (N = 119)** | | | | | |
| *Encoding phase* | | | | | |
| | | Indoor/ outdoor judgment (RTs [ms]) | 901 ± 117 | 891 ± 135 | 845 ± 154 |
| | | Indoor/ outdoor judgment (Error rates [%]) | .6 ± .84 | .9 ± .96 | .8 ± 1.2 |
| *Recognition phase* | | | | | |
| | | d-prime | 1.53 ± .54 | 1.56 ± .51 | 1.49 ± .48 |
| | | Corr. hit rate (%) | 50.2 ± 14.0 | 53.1 ± 14.7 | 51.1 ± 14.9 |
| **fMRI experiment 2: Reward-related memory encoding (N = 63)** | | | | | |
| *Encoding phase (reward task)* | | | | | |
| Monetary condition | | | | | |
| | RTs (ms) | Reward trials | 414 ± 43 | 443 ± 52 | 445 ± 59 |
| | | No-reward trials | 479 ± 50 | 494 ± 64 | 498 ± 58 |
| | Hit Rate (%) | Reward trials | 75.3 ± 3.3 | 76.3 ± 2.9 | 75.0 ± 3.8 |
| | | No-reward trials | 72.3 ± 4.7 | 72.5 ± 4.2 | 73.6 ± 4.5 |
| Social condition | | | | | |
| | RTs (ms) | Reward trials | 448 ± 47 | 471 ± 66 | 452 ± 53 |
| | | No-reward trials | 481 ± 44 | 499 ± 67 | 478 ± 53 |
| | Hit Rate (%) | Reward trials | 73.8 ± 3.4 | 75.4 ± 4.7 | 75.0 ± 3.9 |
| | | No-reward trials | 71.7 ± 5.9 | 74.1 ± 5.1 | 72.2 ± 3.7 |
| *Recognition phase* | | | | | |
| Monetary condition | | | | | |
| | Corr. hit rate (%) | Reward trials | 23.3 ± 11.5 | 15.8 ± 12.3 | 16.3 ± 9.9 |
| | | No-reward trials | 23.0 ± 11.8 | 17.5 ± 13.0 | 22.8 ± 8.8 |
| Social condition | | | | | |
| | Corr. hit rate (%) | Reward trials | 21.8 ± 20.9 | 20.9 ± 10.9 | 21.1 ± 12.1 |
| | | No-reward trials | 22.0 ± 12.9 | 19.7 ± 10.9 | 16.4 ± 14.9 |

Means and standard deviations (SD) are reported (TT: thymine homozygotes, TG: thymine/guanine heterozygotes, GG: guanine homozygotes).

**Table S6:** Brain responses during encoding of novel scenes ($p < .05$, FWE-corrected, minimal cluster size = 10). Abbreviations: BA – Brodmann area

| **Region** | **BA** | **x** | **y** | **z** | **SPM $t_{111}$** | **p (FWE-cor.)** |
|---|---|---|---|---|---|---|
| Left medial frontal gyrus | 11 | -4 | 40 | -16 | 6.71 | <.001 |
| Right middle frontal gyrus | 46 | 46 | 32 | 16 | 8.03 | <.001 |
| Left inferior frontal gyrus | 47 | -32 | 34 | -14 | 10.25 | <.001 |
| | 9 | -42 | 8 | 30 | 13.03 | <.001 |
| Right inferior frontal gyrus | 47 | 30 | 34 | -12 | 9.48 | <.001 |
| | 9 | 42 | 8 | 30 | 10.29 | <.001 |
| Left/Right superior frontal gyrus | 6 | 0 | 10 | 54 | 7.35 | <.001 |
| Left superior frontal gyrus | 6 | -22 | 4 | 56 | 6.97 | <.001 |
| Right precentral gyrus | 6 | 46 | -6 | 58 | 6.75 | <.001 |
| Left precentral gyrus | 4 | -40 | -14 | 58 | 6.59 | <.001 |
| | 4 | -28 | -24 | 56 | 6.40 | <.001 |
| Right parahippocampal gyrus/ hippocampus | 36 | 28 | -42 | -12 | 26.59 | <.001 |
| Left parahippocampal gyrus | 28 | -18 | -14 | -20 | 9.16 | <.001 |
| Right paracentral lobule | 5 | 8 | -42 | 50 | 7.84 | <.001 |
| Left postcentral gyrus | 3 | -34 | -34 | 62 | 6.53 | <.001 |
| Left claustrum/insula | | -26 | 26 | 4 | 7.31 | <.001 |
| Right globus pallidus | | 20 | -2 | -14 | 6.07 | .002 |
| Left thalamus | | -4 | -32 | -6 | 8.20 | <.001 |
| Left/Right cerebellum | | 0 | -54 | -36 | 8.85 | <.001 |
| Left cerebellum | | -18 | -40 | -46 | 6.05 | .002 |

**Table S7:** *RASGRF1* associated brain responses during encoding of novel scenes ($p < .005$, uncorrected for multiple comparisons, minimal cluster size = 10). Abbreviations: BA – Brodmann area

| Region | BA | x | y | Z | SPM $F_{2, 111}$ | p (uncor.) |
|---|---|---|---|---|---|---|
| Right middle frontal gyrus | 9 | 36 | 28 | 30 | 12.10 | <.001 |
| Left middle frontal gyrus | 6 | -26 | 4 | 44 | 9.94 | <.001 |
| | 9 | -38 | 30 | 36 | 7.41 | .001 |
| Left inferior frontal gyrus | 45 | -54 | 28 | 16 | 6.89 | .002 |
| Right prefrontal cortex | | 28 | -8 | 36 | 8.45 | <.001 |
| Left cingulate gyrus | 24 | -8 | -8 | 24 | 13.12 | <.001 |
| | 32 | -16 | 20 | 32 | 10.23 | <.001 |
| | 24 | -16 | -18 | 46 | 9.41 | <.001 |
| | 31 | -22 | -38 | 24 | 7.94 | .001 |
| Right cingulate gyrus | 32 | 18 | 14 | 42 | 7.78 | .001 |
| | 24 | 12 | 0 | 36 | 7.76 | .001 |
| Left parahippocampal gyrus | 34 | -26 | 6 | -22 | 14.11 | <.001 |
| | 19 | -24 | -50 | -12 | 9.12 | <.001 |
| Right hippocampus | | 26 | -10 | -26 | 10.51 | <.001 |
| Left insula | 13 | -44 | -26 | 18 | 9.01 | <.001 |
| Left inferior parietal lobule | 40 | -46 | -34 | 38 | 8.00 | .001 |
| Left superior temporal gyrus | 13 | -44 | -48 | 22 | 8.81 | <.001 |
| Left angular gyrus | 39 | -50 | -68 | 36 | 10.53 | <.001 |
| Left precuneus | 7 | -26 | -68 | 30 | 9.44 | <.001 |
| Right precuneus | 7 | 2 | -60 | 36 | 6.56 | .002 |
| Left occipital cortex | 19 | -22 | -68 | -8 | 7.49 | .001 |
| Left lingual gyrus | 17 | -18 | -86 | -2 | 6.70 | .002 |
| Left midbrain | | -2 | -26 | -4 | 8.65 | <.001 |
| Left putamen | | -34 | -12 | -4 | 7.16 | .001 |
| Right claustrum | | 34 | 18 | 2 | 8.82 | <.001 |
| | | 32 | -20 | 12 | 7.96 | .001 |
| Right thalamus | | 18 | -10 | -2 | 9.49 | <.001 |
| Right caudate | | 10 | 20 | -8 | 12.99 | <.001 |
| Left caudate | | -36 | -10 | -16 | 8.08 | .001 |

**Table S8:** *RASGRF1* associated brain responses during monetary reward DM encoding contrast ($p < .05$, FWE-corrected, minimal cluster size = 10, inclusively masked with the main effect of *RASGRF1* ($p < .05$)). Abbreviations: BA – Brodmann area

| Region | BA | x | y | z | SPM $t_{55}$ | p (FWE-cor.) |
|---|---|---|---|---|---|---|
| Right anterior cingulate | 32 | 3 | 38 | 13 | 4.33 | <.001 |
| Right inferior frontal gyrus | 47 | 42 | 26 | -11 | 4.91 | <.001 |
| Left superior temporal gyrus | 21 | -54 | -25 | -8 | 5.41 | .009 |
| Right fusiform gyrus | 19 | 21 | -82 | -17 | 4.81 | <.001 |
| Left cuneus | 18 | -18 | -100 | 16 | 3.48 | .050 |
| Left putamen | | -18 | 17 | -8 | 4.66 | .002 |

**Table S9:** Potential transcription factor binding sites that are affected by the T/G SNP of *RASGRF1*

| | **T allele** | **G allele** |
|---|---|---|
| ELK1 | x | |
| FOXA1 | x | |
| FOXL1 | x | |
| HNF4A | | X |
| MZF1_1-4 | | X |
| Pdx1 | x | |
| PPARG:RXRA | | X |

Prediction of transcription factor binding sites according to JASPAR database: http://jaspar.cgb.ki.se and to CONSITE: http://asp.ii.uib.no:8090/cgi-bin/CONSITE/consite.

**Table S10:** Behavioral data of the social stimuli experiment

| | **Women** | **Men** | **$t_{29}$ (p)** |
|---|---|---|---|
| N | 16 | 15 | |
| Valence | 3.8 +/- .36 | 3.6 +/- .36 | t = 1.60 (.121) |
| Arousal | 2.6 +/- .82 | 2.2 +/- .88 | t = 1.33 (.194) |
| Dominance | 3.2 +/- .53 | 3.6 +/- .95 | t = -1.40 (.175) |
| Sympathy | 4.6 +/- .41 | 4.4 +/- .40 | t = 1.24 (.227) |
| Attraction | 3.9 +/- .57 | 3.8 +/- .23 | t = .37 (.712) |

Average level +/- standard deviation (SD), t-statistics and *p*- values are shown.

## Supplementary Figures

**Figure S1:** Schematic illustration of fMRI paradigm encoding of novel scenes. Indoor and outdoor scenes were randomized across the run and followed by a black screen with a fixation target. For each picture, participants had to judge whether it is an indoor or outdoor scene. ISI = interstimulus interval. The figure displaying this paradigm has previously been published under Creative Commons license (Schott et al., 2014; see http://www.nature.com/tp/journal/v4/n3/full/tp201410a.html).

**Figure S2:** Schematic illustration of the paradigm of fMRI experiment 2 (reward-dependent memory encoding). Example feedback stimuli are shown for neutral, monetary and social reward trials. Reward and no-reward trials were randomized across the run and followed by a black screen with a fixation target. ISI = interstimulus interval.

**Figure S3:** Prevalence of ametropia and emmetropia. Direct comparison of individuals with myopia only and individuals with no refractive error revealed a trend for a higher prevalence of myopia among T homozygotes ($\chi^2 = 2.99$, $p = .084$).

## References


Bradley, M.M., and Lang, P.J. (1994). Measuring emotion: the Self-Assessment Manikin and the Semantic Differential. *J Behav Ther Exp Psychiatry* 25**,** 49-59.

Schott, B.H., Assmann, A., Schmierer, P., Soch, J., Erk, S., Garbusow, M., Mohnke, S., Pöhland, L., Romanczuk-Seiferth, N., Barman, A., Wüstenberg, T., Haddad, L., Grimm, O., Witt, S., Richter, S., Klein, M., Schütze, H., Mühleisen, T.W., Cichon, S., Rietschel, M., Noethen, M.M., Tost, H., Gundelfinger, E.D., Düzel, E., Heinz, A., Meyer-Lindenberg, A., Seidenbecher, C.I., Walter, H. (2014). Epistatic interaction of genetic depression risk variants in the human subgenual cingulate cortex during memory encoding. *Transl Psychiatry* 4**,** e372. doi: 10.1038/tp.2014.10
