# Supplementary figures and images for: Genetic variation of the RASGRF1 regulatory region affects human hippocampus-dependent memory
Source: Front Hum Neurosci. 2014 Apr 29;8:260. doi: 10.3389/fnhum.2014.00260 (PMC4010733; doi:10.3389/fnhum.2014.00260)

## Supplementary Figure S1

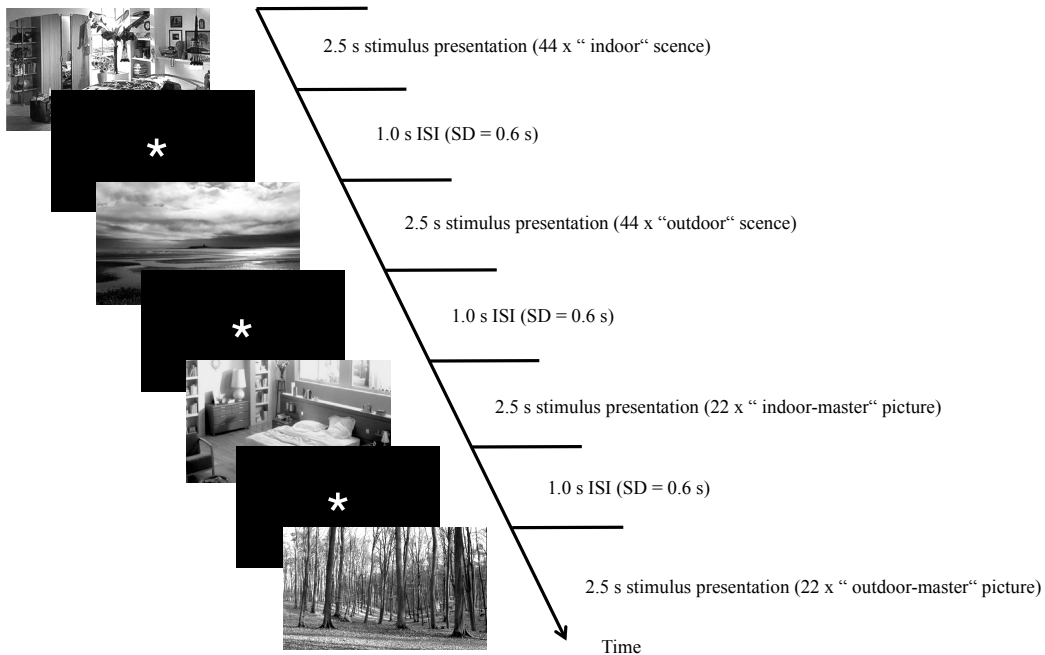

## Supplementary Figure S2

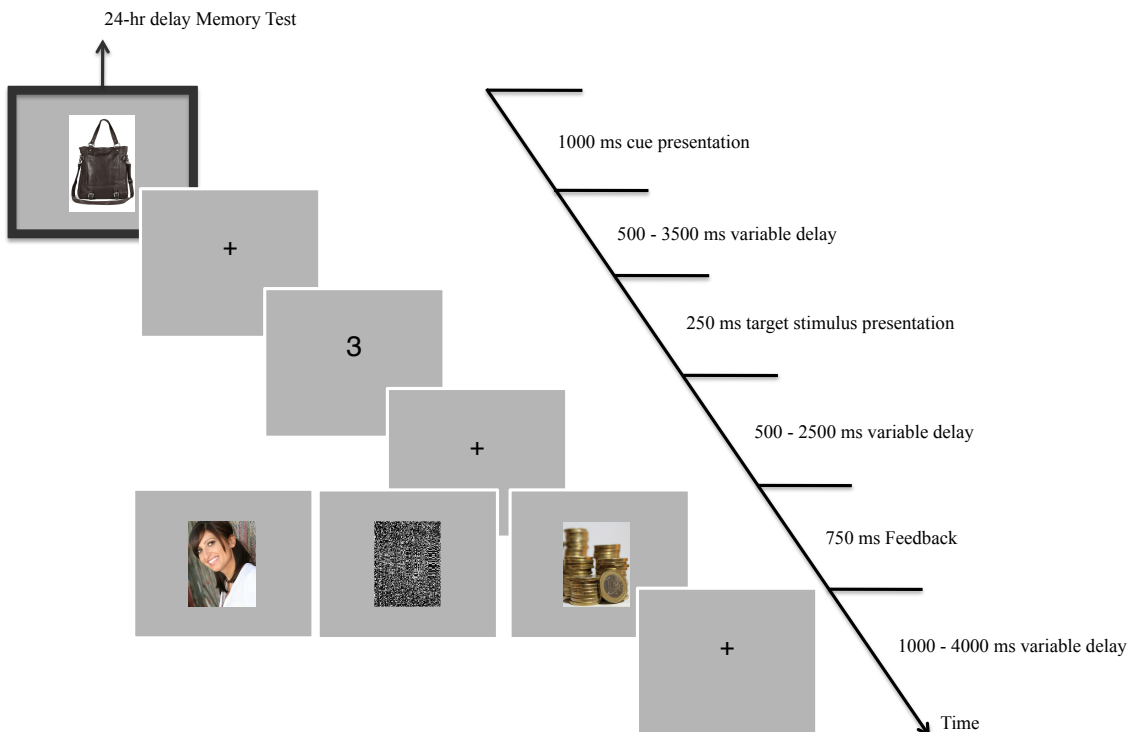

Supplementary Figure S3

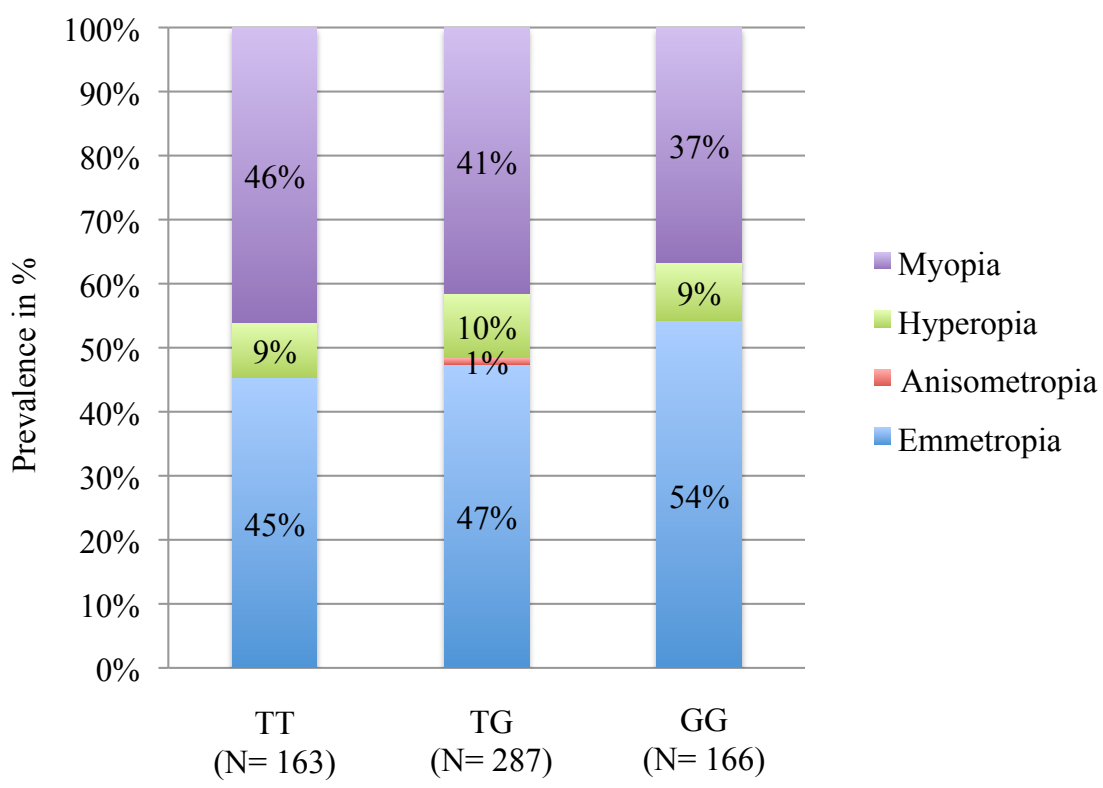

Supplement: Supplementary file 2 [file DataSheet2.PDF]
